# Supplementary material for: Quantitative Analysis of the Potency of Equimolar Two-Drug Combinations and Combi-Molecules Involving Kinase Inhibitors In Vitro: The Concept of Balanced Targeting
Source: Int J Mol Sci. 2021 Sep 3;22(17):9569. doi: 10.3390/ijms22179569 (PMC8430702; doi:10.3390/ijms22179569)
Supplement: Supplementary file 1 [file ijms-22-09569-s001.zip › ijms-1281966-supplementary.pdf]

SUPPLEMENTARY MATERIALS

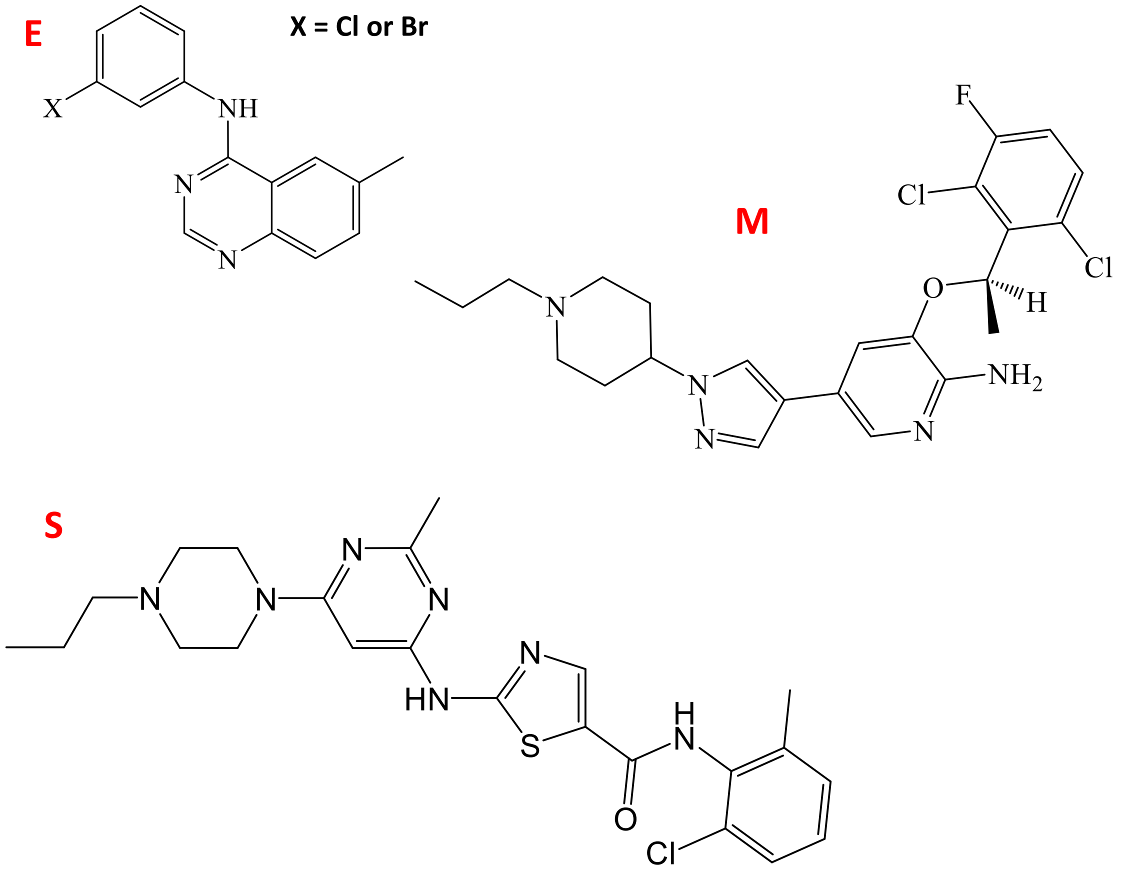

Figure S1. EGFR (E), c-Src (S) and c-Met (M) targeting moieties used in the synthesis of EGFR-c-Src and EGFR-c-Met targeting combi-molecules.

Materials and Methods

Chemistry

^{1}H NMR spectra were recorded on a Varian 300 or 400 MHz spectrometer. Chemical shifts are given as δ values in parts per million (ppm) and are referenced to the residual solvent proton. Mass spectrometry was performed by the McGill University Mass spectroscopy Center and electrospray ionization (ESI) spectra were performed on a Finnigan LC QDUO

spectrometer. Data are reported as m/z (intensity relative to base peak = 100). All chemicals were purchased from Sigma-Aldrich, methyl 6-(chlorocarbonyl)nicotinate from Ellanova Laboratories (Hamden, CT, USA), pyrazine-2,5-dicarboxylic acid from Tyger Scientific Inc. (Ewing, NJ, USA), 5-methoxycarbonylpyridine-2-carboxylic acid from Oakwood Products, Inc. (West Columbia, SC, USA), methyl 5-(chlorocarbonyl)picolinate from AB Chem. Inc. (Dorval, QC, Canada), dasatinib (Sprycel) from Ark Pharmaceutical.

Compound 2a.

To a solution of **1** (0.5 g, 1.86 mmol) with triethylamine (260 µL, 1 eq.) in dry THF (10 mL) at 0 ºC, a solution of freshly chlorinated of 5-methoxycarbonylpyridine-2-carboxylic acid (0.44 g, 1.2 eq.), in dry THF (10 mL) was added dropwise at 0 ºC. The mixture was stirred under argon for 3 h, after which it was evaporated to give a crude powder, which was triturated water. The precipitate obtained was filtered, washed with ethyl acetate and ethyl ether. The brown solid was dried under vaccum to give the intermediate compound (0.76 g) which was used without further purification for the deprotect step. The intermediate compound (0.76 g, 1.75 mmol) was dissolved in dry THF (20 mL), heated slowly to help the dissolution, and potassium trimethylsilanoate (2.24 g, 10 eq.) was added. The mixture was stirred at room temperature under argon for 2h after which it was evaporated to dryness. The resulting solid was triturated in ethyl ether, collected by filtration and redissolved in water. The pH of the solution was adjusted to 2 with HCl 1N and the precipitate obtained was filtered to give a pure brown orange solid **2a** (0.76 mg, 98%). ^{1}H NMR (400 MHz, *DMSO-*d_6) □ ppm 7.39 (d, J=7.83 Hz, 1 H), 7.52 (t, J=7.83 Hz, 1 H), 7.69 (d, J=8.22 Hz, 1 H), 7.89 (br. s., 1 H), 8.01 (d, J=9.00 Hz, 1 H), 8.33 (d, J=8.22

Hz, 1 H), 8.47 (d, J=8.22 Hz, 1 H), 8.56 (d, J=7.83 Hz, 1 H), 8.95 (s, 1 H), 9.21 (br. s., 1 H), 9.24 (br. s., 1 H), 11.35 (s, 1 H), 11.56 (br. s., 1 H), ESI *m/z* 420.1 (MH^+ with ^{35}Cl).

Compound 3a.

To a solution of **2a** (50 mg, 0.12 mmol) and dasatinib (58 mg, 1 eq) in dry DMF (1 mL) were added 1-ethyl-3-(3-dimethylaminopropyl) carbodiimide (EDCI) (25 µL, 1.2 eq.), hydroxybenzotriazole (HOBt) (19 mg, 1.2 eq.) and DMAP (1.5 mg, 0.1 eq.). The mixture was than stirred at room temperature for 24h under argon. The DMF was azeotroped with heptanes to give a crude solid, which was triturated in water. The precipitate was filtered, washed with ethyl ether and dried under vacuum. The resulting brown solid (92 mg) was purified by silica gel chromatography column (THF 100%) to give **3a** (AL660) as a pure powder (40 mg, 38%). ^1H NMR (400 MHz, *DMSO-d_6*□□□ ppm 2.21 (s, 3 H), 2.39 (s, 3 H), 2.55 – 2.63 (m, 4 H), 2.80 (m, 2 H), 3.46 – 3.56 (m, 4 H), 4.50 (m, 2 H), 6.03 (s, 1 H), 7.11 - 7.19 (m, 1 H), 7.21 – 7.31 (m, 2 H), 7.36 – 7.46 (m, 2 H), 7.79 – 7.89 (m, 2 H), 8.03 - 8.13 (m, 1 H), 8.20 (s, 1 H), 8.26 - 8.33 (m, 1 H), 8.36 (d, J=8.21 Hz, 1 H), 8.55 - 8.60 (m, 1 H), 8.61 (s, 1 H), 8.93 (d, J=1.95 Hz, 1 H), 9.22 (d, J=1.56 Hz, 1 H), 9.87 (s, 1 H), 9.94 (s, 1 H), 11.09 (s, 1 H), 11.49 (s, 1 H), ESI *m/z* 889.23 (MH^+ with ^{35}Cl, ^{35}Cl).

Compound 3b.

Compound **3b** (18 mg, 28%) light yellow solid, was synthesized using the same method as compound **3a** using compound **2b** starting with the commercial methyl 5-(chlorocarbonyl)picolinate reagent. ^1H NMR (400 MHz, *DMSO-d_6*□□□ ppm 2.22 (s, 3 H), 2.39 (s, 3 H), 2.54 - 2.63 (m, 4 H), 2.73 - 2.84 (m, 2 H), 3.42 -3.62 (m, 4 H), 4.38 - 4.63 (m, 2 H), 6.04 (s, 1 H), 7.11 - 7.18 (m, 1 H), 7.20 – 7.33 (m, 2 H), 7.33 - 7.46 (m, 2 H), 7.77 - 7.84 (m, 1 H), 7.86 (d, J=8.60 Hz, 1 H), 7.98 - 8.04 (m, 1 H), 8.04 - 8.10 (m, 1 H),

8.20 (s, 1 H), 8.23 - 8.30 (m, 1 H), 8.50 - 8.59 (m, 1 H), 8.62 (s, 1 H), 8.91 (d, J=1.95 Hz, 1 H), 9.29 (d, J=1.56 Hz, 1 H), 9.87 (s, 1 H), 9.99 (s, 1 H), 10.99 (s, 1 H), 11.47 (br. s, 1 H), ESI *m/z* 911.21 (MNa^+ with ^{35}Cl, ^{35}Cl).

Compound 4.

The commercial compound pyrazine-2,5-dicarboxylic acid was firstly chlorinated with an excess of thionyl chloride and a quantitative amount of DMSO. After 24h of reflux under argon, the clear orange mixture was evaporated, well dried under high vacuum and kept under argon at -20°C. Before using it, the solid was dissolved in methylene chloride to remove the unreacted diacid compound, filtered, and dried.

A solution of **1** (50 mg, 0.18 mmol) with triethylamine (180 µL, 7 eq.) in dry CH_2Cl_2/THF (10/2 mL) was added dropwise very slowly via a canula (addition during around 1 hour) to a solution of bischlorocarbonyl linker (76 mg, 2 eq.) dissolved in dry CH_2Cl_2/THF (4/2 mL) at 0 ºC. A precipitate appeared within a few minutes and the mixture was further stirred at room temperature for 1h after the end of the addition, under argon. The orange precipitate was filtered, washed with THF and with water to hydrolyze the acyl chloride (small bubbles can be observed), the brown-orange solid obtained was washed with ethyl ether and dried under vacuum (27 mg). By ^1H NMR, mixture of different compounds was observed: 70% of expected compound **4** with 30% of the dimer and around 30% for starting material pyrazine-2,5-dicarboxylic acid. Without further purification, the mixture was used directly for the last coupling step.

Compound 3c

Compound **3c** was synthesized using the same method as compound **3a,** taking into account the percentage of compound **4** in the mixture estimated by NMR. Compound **3c** was

purified by preparative TLC (silica plate, CH2Cl2/MeOH 9/1) to give a pure yellow solid (13 mg, 8%). ^{1}H NMR (400 MHz, *DMSO-*d_6□□□ ppm 2.22 (s, 3 H), 2.39 (s, 3 H), 2.55 - 2.63 (m, 4 H), 2.75 - 2.85 (m, 2 H), 3.45 - 3.60 (m, 4 H), 4.50 - 4.60 (m, 2 H), 6.04 (s, 1 H), 7.11 - 7.19 (m, 1 H), 7.19 - 7.31 (m, 2 H), 7.35 - 7.45 (m, 2 H), 7.80 - 7.90 (m, 2 H), 8.08 (t, J=1.95 Hz, 1 H), 8.20 (s, 1 H), 8.22 - 8.29 (m, 1 H), 8.62 (s, 1 H), 8.95 (d, J=1.95 Hz, 1 H), 9.32 (d, J=1.17 Hz, 1 H), 9.48 (d, J=1.17 Hz, 1 H), 9.87 (s, 1 H), 9.97 (s, 1 H), 11.19 (s, 1 H), 11.47 (m, 1 H), ESI *m/z* 888.13 (MH^+ with ^{35}Cl, ^{35}Cl).

Compound 6.

To a solution of **7**(40) (22 mg, 0.037 mmol) and **1** (10 mg, 1 eq) in dry DMF (1 mL) were added 1-ethyl-3-(3-dimethylaminopropyl) carbodiimide (EDCI) (7.9 μL, 1.2 eq.), hydroxybenzotriazole (HOBt) (6 mg, 1.2 eq.). The mixture was further stirred at room temperature for 18 h under argon. The DMF was azeotroped with heptanes to give a crude solid which was purified by preparative TLC (silica plate, CH_2Cl_2/MeOH 93/7) gave **8** (AL739) as a pure white powder (11 mg, 35%). ^{1}H NMR (400 MHz, *DMSO-*d_6□□□ ppm 2.22 (s, 3 H), 2.36 (s, 3 H), 2.55 - 2.60 (m, 2 H), 2.62 - 2.74 (m, 4 H), 3.39 - 4.55 (m, 4 H), 4.08 - 4.20 (m, 4 H), 4.37 - 4.50 (m, 2 H), 5.98 (s, 1 H), 7.12 (dd, J=8.01, 1.37 Hz, 1 H), 7.18 - 7.31 (m, 2 H), 7.32 - 7.43 (m, 2 H), 7.71 - 7.81 (m, 2 H), 7.84 (dd, J=8.99, 1.95 Hz, 1 H), 8.02 (t, J=1.95 Hz, 1 H), 8.20 (s, 1 H), 8.53 (s, 1 H), 8.71 (d, J=1.56 Hz, 1 H), 9.86 (s, 1 H), 9.90 (s, 1H), 10.38 (s, 1 H), 11.43 (br s, 1 H), ESI *m/z* 840.23 (MH^+ with ^{35}Cl, ^{35}Cl).

Compound 10.

The amino compound **9** (aminoquinazoline synthesized as described (41) RB10) (1 g) was dissolved in dry acetonitrile (40 mL) under argon and, cooled to -5°C. Nitrosonium

tetrafluoroborate (2 eq.) in acetonitrile was added directly. After 30 min at -5°C, the resulting solution was added dropwise to another solution of 1-piperazineethanol (4 eq.) in acetonitrile with triethylamine (4 eq.) at 0°C, after which the mixture was extracted with dichloromethane and brine. The organic layer was dried with magnesium sulfate and evaporated to provide a brown residue, which was purified by silica gel chromatography (CH_2Cl_2/MeOH 9/1) to give the compound **12** as a pure product (354 mg, 24.5%). ^{1}H NMR (300 MHz, *DMSO-d_6*) □ ppm 2.51 (m, 2H), 2.63 (br s, 4 H), 3.55 (q, *J = 6.1 Hz*, 2 H), 3.81 (t, *J = 5 Hz*, 4 H), 4.50 (t, *J = 5 Hz*, 1 H), 7.30 (m, 2 H), 7.77 (d, *J = 9 Hz*, 1 H), 7.95 (m, 2 H), 8.26 (t, *J = 1.9 Hz*, 1 H), 8.47 (d, *J = 1.8 Hz*, 1 H), 8.59 (s, 1 H), 9.89 (s, 1 H).

Compound 11.

To a solution of compound **10** (353 mg) with pyridine (2 eq.) and trimethylamine (3 eq.) in dry dichloromethane (10 mL) was added dropwise 2-bromorthylchloroformate (2 eq.) at 0°C. The mixture was further stirred at room temperature for 1h and evaporated. The crude product obtained was purified by silica gel chromatography (CH_2Cl_2/MeOH 9/1) to give the compound **11** as a pure product (161 mg, 34.3%). ^{1}H NMR (300 MHz, *DMSO-d_6*) □ ppm 2.68 (m, 6 H), 3.70 (t, *J = 6 Hz*, 2 H), 3.82 (t, *J = 4.8 Hz*, 4 H), 4.25 (t, *J = 5.4 Hz*, 2 H), 4.39 (t, *J = 6 Hz*, 2 H), 7.30 (m, 2 H), 7.77 (d, *J = 8.8 Hz*, 1 H), 7.95 (m, 2 H), 8.26 (t, *J = 1.9 Hz*, 1 H), 8.47 (d, *J = 1.8 Hz*, 1 H), 8.59 (s, 1 H), 9.89 (s, 1 H).

Compound 12 (LP121).

To a solution of **11** (152 mg) in dry DMF (2 mL) was added potassium iodide (1.5 eq.), trimethylamine (3 eq.) and commercial crizotinib (1.1 eq.). The mixture was stirred under argon at 40°C during 4 days. The DMF was azeotroped with heptanes to give a crude solid, which was purified by silica gel chromatography (CH_2Cl_2/MeOH 9/1) to give the

compound **12** as a pure orange powder (93 mg, 38%). ^{1}H NMR (300 MHz, *DMSO-*d_6) □ ppm 1.76 (d, *J = 6.7 Hz*, 3 H), 1.90 (m, 4H), 2.14 (m, 2H), 2.65 (m, 8 H), 2.95 (d, *J = 10.8 Hz*, 2 H), 3.81 (t, *J = 5 Hz*, 4 H), 4*.08(m,* 1 H), 4.21 (m, 4 H), 5.63 (m, 2 H), 6.04 (q, *J = 6.9 Hz*, 1 H), 6.86 (d, *J = 1.8 Hz*, 1 H), 7.29 (m, 2 H), 7.40 (m, 1H), 7.56 (s, 1H), 7.53 (m, 1H), 7.75 (m, 2 H), 7.95 (m, 3H), 8.26 (t, *J = 1.8 Hz*, 1 H), 8.46 (d, *J = 2.1 Hz*, 1 H), 8.59 (s, 1 H), 9.88 (s, 1 H), HRMS: m/z calcd for $C_{44}H_{46}BrCl_2FN_{12}O_4.H^+$ 975.23822; found 975.23657 (Δ = -1.69 ppm).

Results

Chemistry

The synthesis of the first series of compounds **3a-b (AL660-690)** and **3c (AL692)** proceeded according to Scheme 1 and 2 respectively. The aminoquinazoline **1 (42)** was treated with an excess of the appropriate chlorocarbonyl linker to give protected intermediates, whose methyl ester was removed in the presence of potassium silanoate base to give acid **2a-b** (Scheme 1). After the acid **2a-b** was coupled with dasatinib in the presence of EDCI, HOBt and DMAP gave **3a-b** (AL660 and AL690).

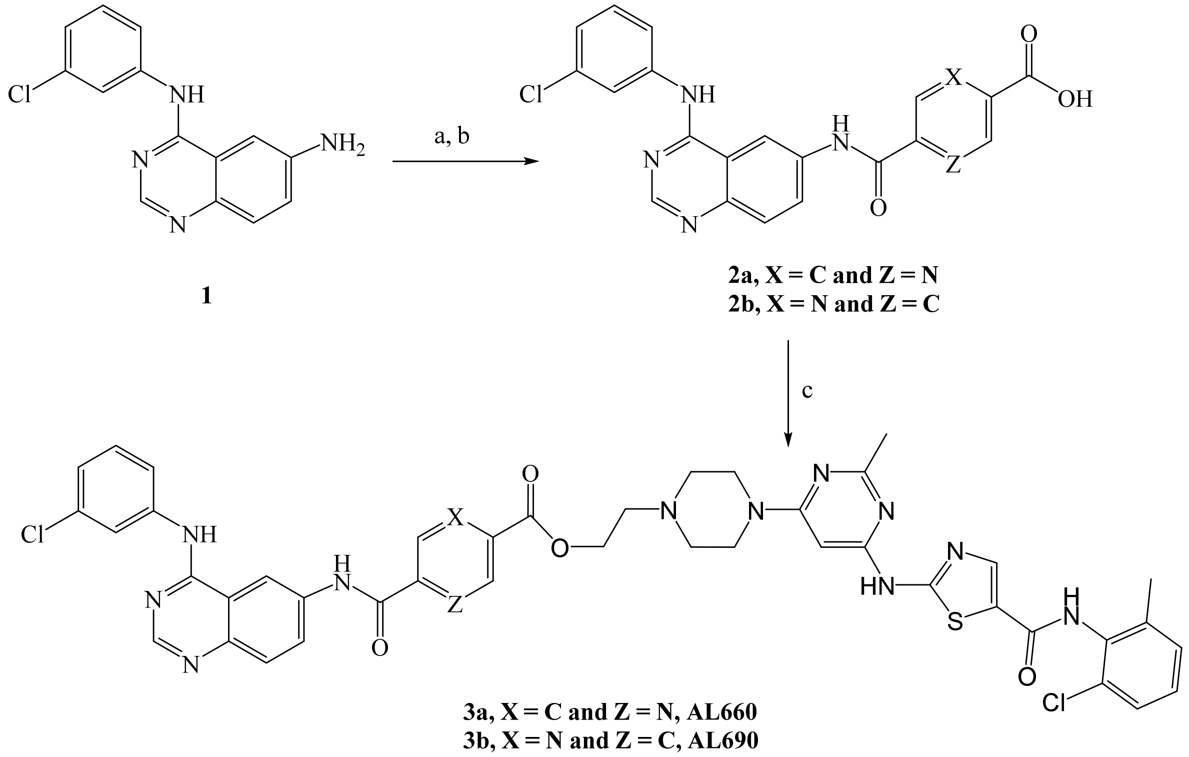

Scheme S1. a) methyl 6-(chlorocarbonyl)nicotinate or methyl 5-(chlorocarbonyl)picolinate, Et_3N, THF, 0°C, 3 h; b) Me_3SiOK, THF, rt, 2 h; c) dasatinib, EDCI, HOBt, DMAP, DMF, rt, 24 h.

Compound **3c** was synthesized following the same method (Scheme 2), only the linker pyrazine-2,5-dicarboxylic acid was commercially available so this diacid reagent was before chlorinated by refluxing in thionyl chloride with catalytic DMSO during 24h. The aminoquinazoline **1** was treated with an excess of the bischlorocarbonyl linker and triethylamine to obtain a not selective coupling reaction, the optimal conditions gave 70% of expected compound **4** with 30% of dimer and also still a presence of starting reagent pyrazine-2,5-dicarboxylic acid. This mixture was directly used to be coupled with dasatinib

in the presence of EDCI, HOBt and DMAP to give the final compound **3c (AL692)** with a very low yield.

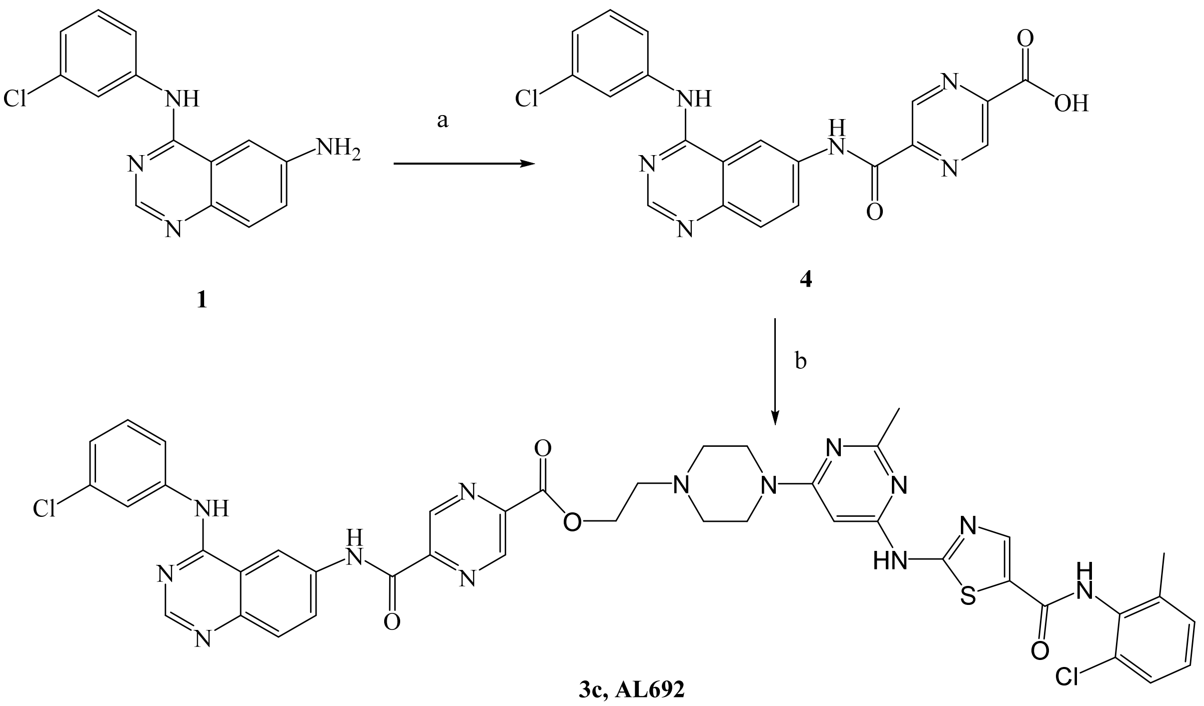

Scheme S2. a) pyrazine-2,5-dicarboxylic acid, $SOCl_2$, DMSO, reflux, 24 h and Et_3N, CH_2Cl_2/THF, 0°C, 1 h; b) dasatinib, EDCI, HOBt, DMAP, DMF, rt, 24 h.

The synthesis of the compounds **6** and **7** proceeded according to Scheme 3 and was already published in Rao *et al.* 2015 (12). Briefly, dasatinib was treated with an excess of succinic anhrydride to give the compound **7**, which was coupled with **1** or 8 (AL621) (43) in the presence of EDCI, HOBt and gave respectively compound **8** (AL739) and **10** (AL776) (40).

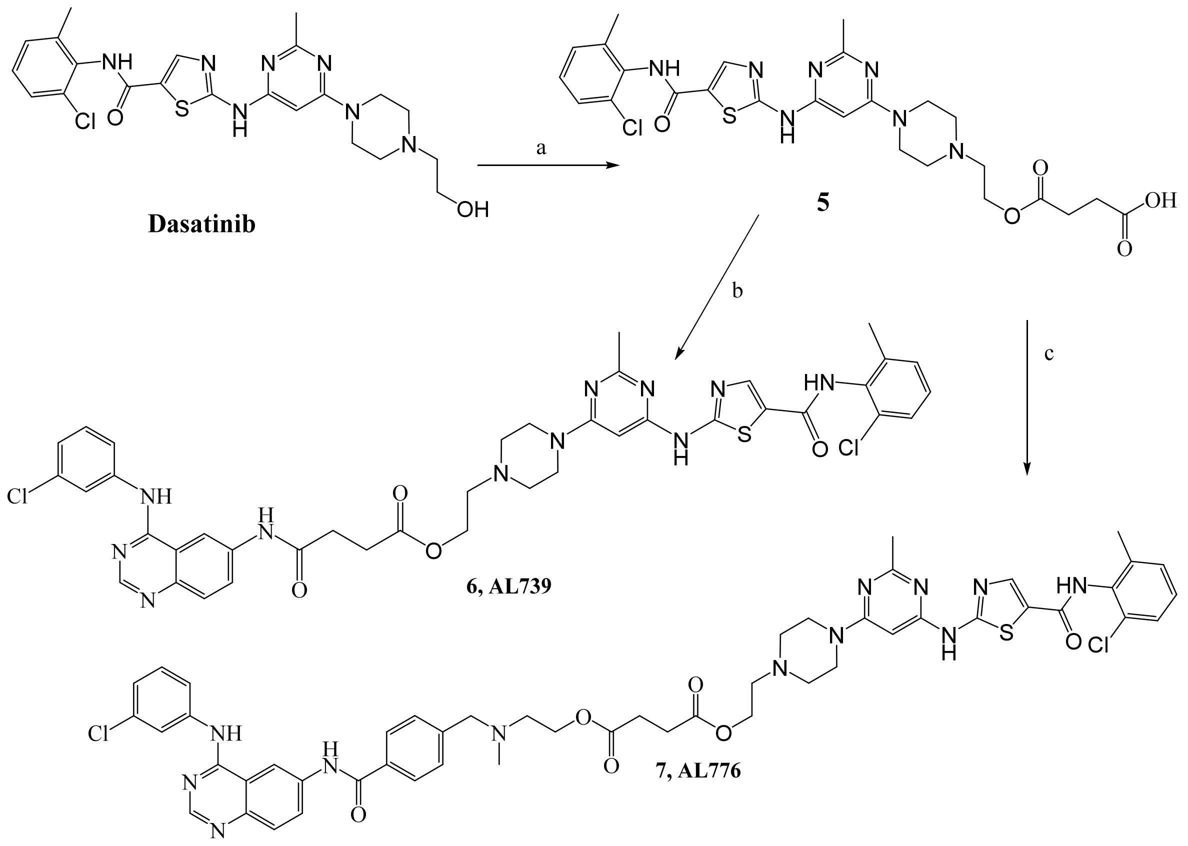

Scheme S3. a) succinic anhydride, DMAP, DMF, 50°C, 18 h; b) compound 1, EDCI, HOBt, DMAP, DMF, rt, 18 h; c) compound **8** (AL621), EDCI, HOBt, DMAP, DMF, rt, 18 h.

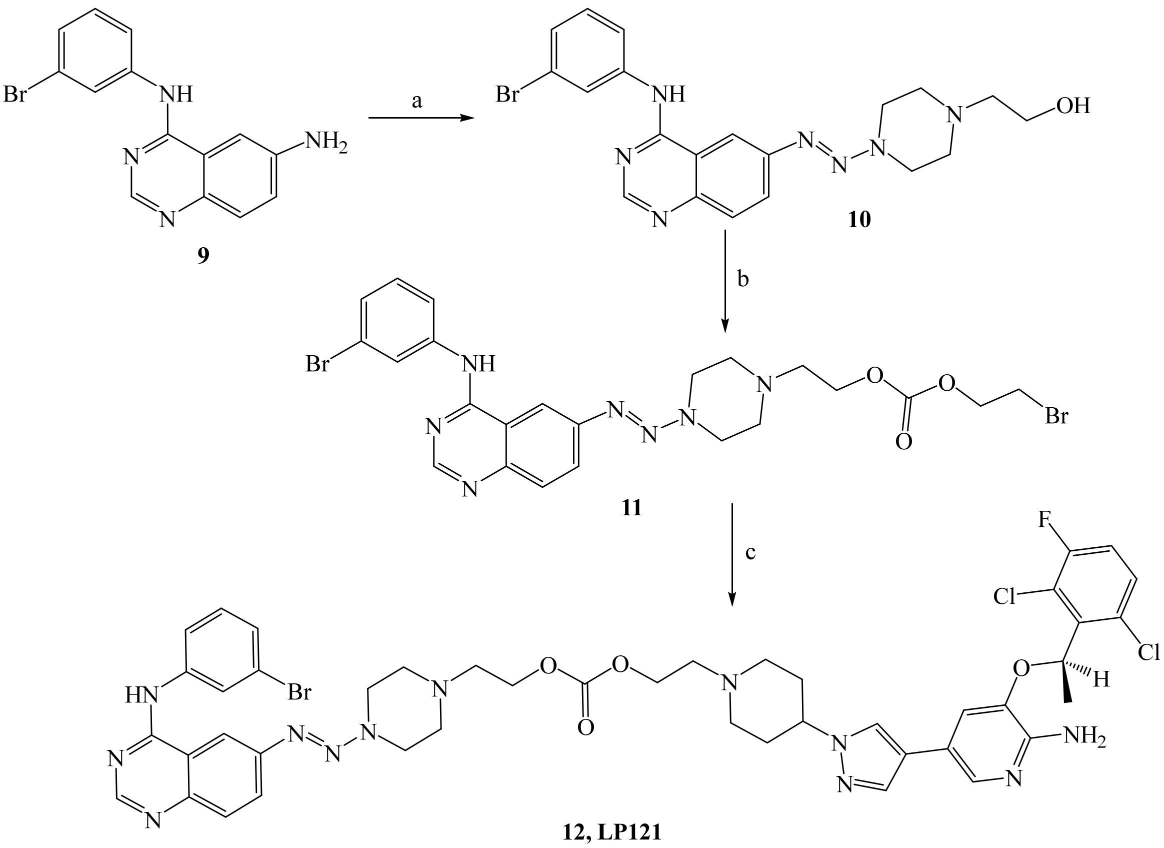

Scheme S4. a) NOBF4,1-piperazineethanol, Et_3N, ACN, -5°C, 1 h; b) 2-bromoethylchloroformate, pyridine, Et_3N, CH_2Cl_2, 0°C to rt, 1 h; c) crizotinib, KI, Et_3N, DMF, 40°C , 4d.

The synthesis of the compounds **12 (LP121)** proceeded according to Scheme 5. The aminoquinazoline **9** (44) was diazotized in dry acetonitrile with nitrosonium tetrafluoroborate to provide the diazonium salt that was coupled with 1-piperazineethanol in the presence of triethylamine *in situ*. The alcohol compound **10** obtained was treated with the commercial 2-bromorthylchloroformate to give intermediate **11**, which was coupled with crizotinib in the presence of potassium iodide and trimethylamine to give the final compound **12**.
